# Role of Envelopment in the HEV Life Cycle

**DOI:** 10.3390/v8080229

**Published:** 2016-08-18

**Authors:** Xin Yin, Xinlei Li, Zongdi Feng

**Affiliations:** 1 Center for Vaccines and Immunity, The Research Institute at Nationwide Children’s Hospital, Columbus, OH 43205, USA; xin.yin@nationwidechildrens.org (X.Y.); xinlei.li@nationwidechildrens.org (X.L.); 2 Department of Pediatrics, The Ohio State University College of Medicine, Columbus, OH 43205, USA

**Keywords:** hepatitis E virus, quasi-envelopment

## Abstract

Hepatitis E virus (HEV), an enterically transmitted hepatotropic virus, was thought to be non-enveloped for decades. However, recent studies have revealed that the virus circulating in the patient’s blood is completely cloaked in host membranes and resistant to neutralizing antibodies. The discovery of this novel enveloped form of HEV has raised a series of questions about the fundamental biology of HEV and the way this virus, which has been understudied in the past, interacts with its host. Here, we review recent advances towards understanding this phenomenon and discuss its potential impact on various aspects of the HEV life cycle and immunity.

## 1. Introduction

Hepatitis E virus (HEV) infection is a major cause of acute hepatitis worldwide [1]. HEV is classified as the sole member of the *Orthohepevirus* genus within the *Hepeviridae* family. At least four genotypes are responsible for the diseases in humans [2,3]. Genotypes 1 and 2 only infect humans and are mainly responsible for large waterborne outbreaks in developing countries [3,4]. Genotypes 3 and 4 are autochthonous in industrialized countries [2], where infections are zoonotic [5]. HEV infections are usually self-limited but can cause serious liver diseases including fulminant hepatitis. In addition, persistent infections with genotype 3 HEV have been reported in immunocompromised patients and can lead to a rapid progression to liver cirrhosis [6,7]. Infectious HEV has also been found in the blood supply, raising the possibility of blood-borne transmission [8].

This small RNA virus has been considered non-enveloped since its discovery 25 years ago [9,10]. However, virions in feces are naked, and recent studies have shown that those circulating in the bloodstream are cloaked in a host cell membrane [11]. These novel virus particles are infectious, yet they do not carry viral antigens on the surface and are completely resistant to neutralizing antibodies in standard neutralization assays. This dual life style is similar to that of hepatitis A virus (HAV), another hepatotropic virus that is phylogenetically unrelated to HEV. We recently discovered that HAV exists in the blood in an enveloped form, but it is shed in the feces as naked virions [12]. The discovery of these novel enveloped virus particles, termed “quasi-enveloped” virions, has raised a series of questions about the fundamental biology of these infections. In this review, we focus on HEV and discuss the potential impact of this envelopment on the HEV life cycle.

### Envelopment of HEV: A Novel Mechanism for Non-Cytolytic Virus Release and Immune Evasion

HEV was initially isolated from patient stool and visualized by immunoelectron microscopy as non-enveloped particles [13]. The virions are 27–34 nm in diameter with an isosahedral morphology. The capsid structure is similar to that of caliciviruses, and because of that HEV was initially misclassified as a member of the *Caliciviridae* family. With molecular cloning of the HEV genome in 1990 [14], it was recognized that the capsid is composed of a single viral protein encoded by the viral open reading frame (ORF) 2 gene. Each capsid consists of 180 copies of ORF2 arranged as an icosahedron with T = 3 symmetry [15]. Based on sequence and structural similarities to other viruses, three domains have been assigned to the capsid: the shell (S) domain, the middle (M) domain, and the protruding (P) domain. The S domain forms the continuous capsid shell, and is the most conserved region among HEV genotypes. The P domain is the major target for neutralizing antibodies, and contains a putative cell surface receptor binding domain [16,17,18,19,20].

The naked HEV virions found in feces have a buoyant density of approximately 1.27 g/mL. However, Takahashi and colleagues found HEV in the culture supernatants exhibited a significantly lesser density (about 1.15 g/mL) [21]. Subsequent studies demonstrated that HEV particles circulating in the blood or culture supernatants are wrapped in host-derived membranes [21,22,23]. These membrane-associated HEV particles lack detectable viral proteins on the surface and are highly resistant to neutralizing antibodies in the serum; hence, they are termed quasi-enveloped HEV, or eHEV [11]. From an evolutionary point of view, the envelopment of HEV in a host membrane could offer several advantages to HEV. Non-enveloped viruses are typically released by lysing infected cells, which unleashes immunostimulatory danger signals from virus-infected cells. By cloaking itself in a host-derived membrane during exit, HEV is capable of exiting cells non-cytolytically, as evidenced by the absence of the cytopathic effects of HEV-infected cells [13]. By avoiding cell lysis, HEV has reduced the level of danger signals.

Moreover, because the host membrane completely masks the viral antigens, antibodies are unable to bind and neutralize the circulating virus particles. Notably, the vast majority of, if not all, virus particles released from cultured cells infected with HEV are enveloped, and cannot be precipitated or neutralized by convalescent human serum, unless the virions are first disrupted with detergent [11,24]. The release of eHEV from culture cells has allowed detailed analyses of the mechanism for HEV envelopment and its impact on the virus life cycle.

## 2. Biogenesis of Enveloped HEV Particles

**Figure 1:**
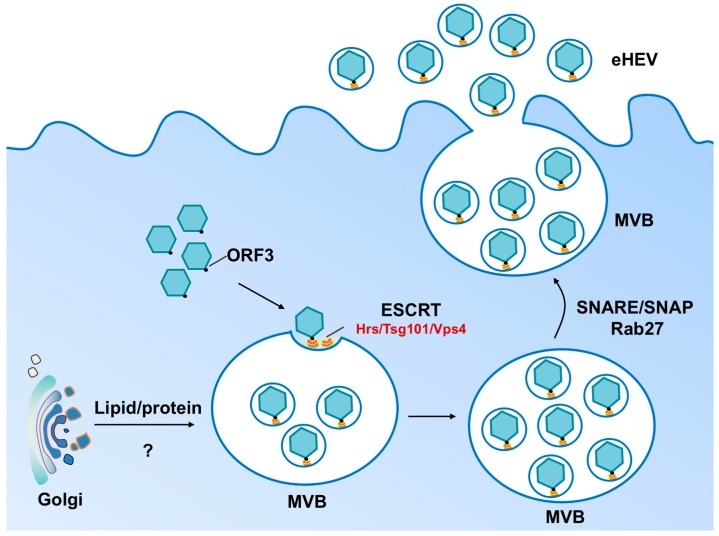
Model for biogenesis of eHEV. During the late stage of infection, HEV capsids are assembled in the cytoplasm and encapsidate viral genomes. ORF3 interacts with both Tsg101 (an ESCRT-associated protein) and the viral capsid, directing the budding of the viral capsid into the multivesicular bodies (MVB). Other components of the ESCRT machinery, including Hrs and Vps4, are also implicated in eHEV release. The quasi-enveloped HEV particles are subsequently released from infected cells upon fusion of the MVB membrane and the plasma membrane, a process that is regulated by Rab27 and other host factors such as SNARE/SNAP [31]. The origin of the eHEV membrane remains unclear, but is likely to derive from internal organelles such as the Trans-Golgi network.

The 7.2 kb positive-sensed HEV genome encodes three proteins: ORF1, ORF2, and ORF3. ORF1 is a large nonstructural protein involved in HEV RNA replication. ORF2 is a 72 kD capsid protein required for virus assembly and receptor binding. A third viral protein, ORF3, is a small multifunctional protein essential for virus egress. The C-terminus of ORF3 contains a conserved PSAP (i.e., amino acids proline, serine, alanine, and proline) late domain motif, through which ORF3 interacts with the tumor susceptibility gene 101 (Tsg101) protein [25,26,27]. Tsg101 is a component of the cellular endosomal sorting complexes required for transport (ESCRT) machinery involved in the budding of many enveloped viruses [28]. This interaction likely promotes the budding of newly assembled HEV virions into multivesicular bodies (MVB). If so, the enveloped viral particle in the MVB would likely be released from the cell upon fusion of the MVB membrane with the plasma membrane (Figure 1). Consistent with this model, Rab27 and Hrs, components required for exosomal secretion, as well as the vacuolar protein sorting-associated protein 4 (Vps4), an ATPase that strips ESCRT components from the membrane, are required for HEV egress [26,29]. Furthermore, CD63 and CD81, ubiquitous markers for exosomes, have been shown to associate with quasi-enveloped HEV virions [29], further suggesting that HEV hijacks the exosomal pathway for egress. In agreement with its essential role in mediating HEV release, ORF3 is found in eHEV but not in the non-enveloped HEV particles [21].

Although the current evidence suggests that HEV release is dependent on the cellular ESCRT machinery, the origin of the eHEV membrane remains uncertain. One recent study concluded that the membrane is derived from the trans-Golgi network (TGN) because it contains the trans-Golgi network protein 2 (TGOLN2) [30]. Since TGOLN2 cycles between the TGN and endocytic compartments, it is possible that this membrane protein is transferred to the MVB via vesicular transport and is “accidentally” incorporated into the eHEV envelope. Determination of the complete protein and lipid composition and quantity of the eHEV membrane components and its comparison to exosomal membranes is likely to yield a clearer understanding of the origin of the eHEV membrane and the mechanism of HEV envelopment.

## 3. Impact of Envelopment on HEV Cell Entry and Spread

Given the differences between eHEV and HEV, it is likely that the two forms use different mechanisms to enter target cells. For non-enveloped HEV, its initial cell attachment is thought to be mediated through heparan sulfate proteoglycans [32], which are expressed on the surface of many cell types and involved in the attachment of both enveloped and non-enveloped viruses [33,34]. However, while non-enveloped HEV infection is sensitive to competition with soluble heparan sulfate, eHEV infection is not [35]. Cell attachment of eHEV is also less efficient when compared to non-enveloped virions as evidenced by delayed binding kinetics, most likely due to the lack of specific virus-cell interactions. In agreement with this in vitro observation, serum- or cell culture-derived HEV, which is enveloped, is less infectious than fecal virus, which is non-enveloped, when tested in uPA-SCID mice transplanted with human liver cells [36,37].

**Figure 2:**
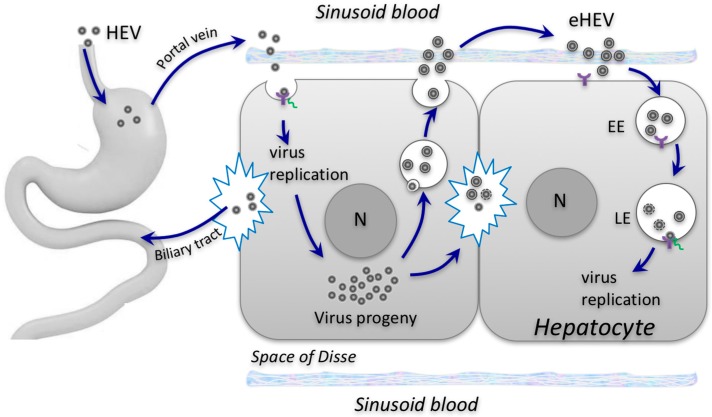
Proposed model for HEV entry and spread. Non-enveloped HEV virions (HEV) are acquired orally and enter the bloodstream via an unknown mechanism. They infect, propagate, and are then released from the basolateral (sinusoidal) sides of hepatocytes into the bloodstream as quasi-enveloped virions (eHEV). eHEV mediate subsequent rounds of infection within the liver. Virions are also released from the apical (bile canaliculi) side of infected hepatocytes and excreted via the biliary tract into the feces. Non-enveloped and enveloped HEV particles enter cells via distinct pathways, but likely use the same putative cellular receptor (purple) that is present both at the cell surface and on the endosomal membrane. EE, early endosome. LE, late endosomes. N, nucleus.

Following cell attachment, both non-enveloped and enveloped HEV virions are internalized through a clathrin- and dynamin-2-dependent pathway [38,39]. However, the subsequent steps differ. While entry of eHEV is critically dependent on early-late endosomal trafficking and acidification, non-enveloped HEV is not. Furthermore, blocking lipid degradation in the lysosome, either by depleting Niemann-Pick disease type C1 (NPC1) proteins or treating cells with an inhibitor of the lysosomal acid lipase (LAL), reduces the entry of eHEV, but not non-enveloped HEV [35]. These results suggest that the eHEV membrane is degraded in the endolysosome, a step that would reveal the capsid, allowing it to bind to its receptor. Therefore, the site of penetration of non-enveloped and enveloped HEV particles appears to be different: eHEV likely penetrates in the endolysosome, but HEV’s penetration of cells likely occurs at or in the vicinity of the plasma membrane (Figure 2).

## 4. Impact of Envelopment on HEV Transmission between Hosts

HEV is primarily transmitted enterically. Following ingestion, HEV penetrates the gut through a poorly defined mechanism. Although the human gut stands as a likely first target for HEV, there is no compelling evidence that HEV enters and replicates in human gut cells. Subsequently, HEV enters the bloodstream and is carried to its main target organ, the liver, where the virus multiplies within hepatocytes. Newly synthesized virions are released at both the apical (canaliculous) and the basolateral (sinusoidal) surfaces of infected hepatocytes. While some are released from the basolateral membrane (in the form of eHEV) into the sinusoidal blood and re-enter the circulation system, the bulk of the virus is believed to be released from the apical surface into the bile canaliculi, where it enters the biliary tract and is subsequently shed in feces.

Only non-enveloped HEV virions are detected in bile and feces, but the form of the HEV virion that is released from the apical surface of the hepatocyte is likely the enveloped HEV. Intriguingly, recent studies using humanized mice have shown that ORF3 is predominantly localized to the bile canaliculi in HEV-infected liver, suggesting that the eHEV particles are released from the apical membrane [36,37]. This possibility is consistent with a study showing that the predominant localization of ORF3 is at the apical membrane in HEV-infected Caco2 cells (a human intestinal cell line) [40]. eHEV released from the apical, canalicular membrane of the hepatocyte would enter the bile. Incubation of eHEV with human bile results in an increase in the eHEV density [41], suggesting that the detergent action of bile may degrade the eHEV membrane, resulting in non-enveloped HEV in feces.

In addition to the fecal-oral route, transfusion creates a potential route of HEV transmission from person to person [42]. Infectious HEV has been found in blood components, and has caused persistent infection in immunosuppressed patients receiving blood transfusions [6,43,44]. However, as mentioned above, recent studies have found that serum-derived HEV is less infectious for chimeric mice compared to the fecally derived HEV [37,45]. Since the HEV particles in the culture medium and serum are coated with host membrane, the envelope likely reduces its attachment to permissive cells, thereby reducing the risk of infection [35].

## 5. Impact of Envelopment on Extrahepatic Manifestation Associated with HEV Infection

Several extrahepatic symptoms have been associated with HEV infection, including neurologic [46], renal [47] and rheumatologic manifestations [48]. Both HEV RNA and antigens were detected persistently in the urine of a patient with chronic HEV infection [47], suggesting that HEV might replicate in the kidney and account for the observed kidney dysfunction. HEV RNA and antigens were detected in the cerebrospinal fluid [49]. Moreover, HEV is able to infect and replicate in human placental cells and neuronal-derived cells [46,50]. Although compelling evidence for HEV replication in these tissues is still lacking, a broad host cell range may be facilitated by the cloaking of HEV in the host cell membrane [11,29]. Because of its exosome-like quasi-envelope in the blood, eHEV could be taken up by different cell types through an endocytic process that does not depend on viral receptors, though it is not clear that this process would result in infection. In addition, similarities between the membrane-encased HEV virion and exosomes suggest the possibility that eHEV may penetrate immunologically privileged sites such as the central nervous system (CNS) as do exosomes [51,52].

## 6. Conclusions

The discovery of the enveloped form of HEV in circulation has fundamentally changed concepts about the mechanism of virus infection and pathogenesis. Given the lack of specific treatment for HEV, a better understanding of the role of envelopment in HEV infection may help identify targets for therapeutic intervention. Future studies may lead to a better understanding of the origin and structural components of the eHEV envelope, and the host factors/pathways involved in the eHEV release and re-entry into cells. With the improvement of cell culture systems and the recently developed humanized chimeric mouse model, it is expected that many details surrounding this novel phenomenon will be revealed in the near future.
